# Knowledge, Attitude and Practice toward Infant Oral Healthcare among the Pediatricians of Mysore: A Questionnaire Survey

**DOI:** 10.5005/jp-journals-10005-1315

**Published:** 2015-09-11

**Authors:** MD Indira, Kanika Singh Dhull, B Nandlal

**Affiliations:** Senior Lecturer, Department of Pedodontics and Preventive Dentistry, JSS Dental College, JSS University, Mysore, Karnataka, India; Reader, Department of Pedodontics and Preventive Dentistry, Kalinga Institute of Dental Sciences, Bhubaneswar, Odisha, India; Professor, Department of Pedodontics and Preventive Dentistry, JSS Dental College, JSS University, Mysore, Karnataka, India

**Keywords:** Infant oral healthcare, Pediatricians, Preventive dentistry.

## Abstract

**Background:** The aim of this study was to study the knowledge, attitude and practice of the pediatricians toward infant oral healthcare and the objective was to determine what can improve the knowledge, attitude and practice toward infant oral healthcare.

**Materials and methods:** A systematic random survey of pediatricians in Mysore received a questionnaire pertaining to individual details, knowledge level and approach toward infant oral healthcare.

**Results:** Most of pediatricians acknowledged the importance of pediatric dentistry. Pediatricians agree that it is important to do dental examination before 1 year. The importance of initiating oral hygiene practice before the eruption of first tooth was not seen to be prevalent among the pediatricians. Most of them were less aware of the first dental visit including early childhood caries (ECC). All pediatricians agree that both medical and dental professionals together are responsible for infant oral healthcare. They should work together to appropriately educate and train themselves to be able to provide risk assessment and to provide preventive oral health services.

**How to cite this article:** Indira MD, Dhull KS, Nandlal B. Knowledge, Attitude and Practice toward Infant Oral Healthcare among the Pediatricians of Mysore: A Questionnaire Survey. Int J Clin Pediatr Dent 2015;8(3):211-214.

## INTRODUCTION

Early childhood caries (ECC) is the most prevalent infectious disease and major threat to oral health in infants and children as reported by center for disease control and prevention and the National institute of health.^1^ Early childhood caries and the more severe form of ECC (S-ECC) are particularly virulent forms of caries, beginning soon after tooth eruption; developing on smooth surfaces, progressing rapidly, and having a lasting detrimental impact on the dentition.^2^ It is a lifestyle disease with the biological, behavioral and social discriminates. American Academy of Pediatrics Dentistry (AAPD) recognizes that ECC emerges in all the cultural and economic pediatric population.^3^ Alhough ECC is preventable, more than 50% of the children have caries by the time they reach the kindergarten.^4^

In 1986, American Academy of Pediatric Dentistry adopted guidelines on infant oral health as a way to promote oral health and prevent oral disease in very young children.^5^ The 25th anniversary of this AAPD policy statement offers an opportunity to rededicate ourselves and increase our efforts to assure equity of oral health for all the children.^6^ Indian Society of Pediatric and Preventive Dentistry (ISPPD) also strives to achieve this goal with the motto ‘every child has the fundamental right to his/her total oral health’.^7^ The AAPD recognizes that infant oral health is one of the foundations upon which preventive education and dental care must be built, to enhance the opportunity for a lifetime free from preventable oral disease. It proposes recommendations for preventive strategies, oral health risk assessment, anticipatory guidance and therapeutic interventions to be followed by dental, medical, nursing, and allied health professional programs.^8^ It also states that dental-caries risk assessment, based on a child’s age, biological factors, protective factors, and clinical findings, should be a routine component of periodic examinations by oral health and medical providers.^9^

Promotion of oral health and preventive dental care are fundamental concepts in pediatric dentistry. Today, pediatric dentistry possess a body of scientific knowledge and technology to assist parents in raising caries-free children. An early screening of children below 1 year of age is an excellent opportunity for the detection of risk factors. The goal is to provide infants and toddlers with a pleasant, non-threatening introduction to dentistry and to establish and reinforce the foundation for sound dental habits. There is a need to move away from the surgical approach of managing oral diseases and embrace the concept of oral care beginning at infancy or even prenatally.

Since children less than 3 years are not seen routinely by the dentist, they are at risk of developing dental disease. Pediatricians who see a child from birth as part of well baby visit program are in the best position to identify early dental problems and to educate the parents about the early oral preventive healthcare. They also can provide screening services for early detection of dental disease, provide advice about the need to seek dental care and refer those children in need to pediatric dentist. A key element of comprehensive care for children thus involves the coordination of services between medical and dental providers so that they can provide appropriate services at the appropriate ages.

One of the factors which affect the performance of preventive dentistry is the knowledge, attitude and practice of the pediatrician concerning this issue. Basic questions about the delivary of infant oral healthcare, dental referral process and its outcomes remain unanswered. Thus, the present study is designed with the objective to gather the data and evaluate the level of knowledge, attitude and practice toward infant oral healthcare among the pediatricians.

## MATERIALS AND METHODS

The present survey was undertaken among the pediatricians of Mysore, as listed in the Indian Academy of Pediatrics (IAP), Mysore chapter A comprehensive questionnaire was prepared based on the studies done by Tegwyn et al, Prakash et al and Murthy et al.^10-12^

The questionnaire contained 27 questions. To check the reliability and validity of the questionnaire, a pilot study was done among 10 pediatricians. The results obtained were subjected to statistical analysis. Cronbach’s alpha value of 0.84 showed a good internal consistency of the questionnaire. After the questions were considered to be reliable, it was handed over personally to all the pediatricians who agreed to participate in the survey. The questionnaire was collected after 1 day. The results obtained were analyzed using statistical package for social science (SPSS) software descriptive and inferential statistical tests.

## RESULTS

Among a total of 120 pediatricians as listed in the IAP, Mysore chapter, 97 of them completed the questionnaire, giving the response rate of 80.83%. The results of the study are tabulated and discussed.

## DISCUSSION

The IAP Mysore chapter lists 120 pediatricians, 97 of which responded for the study giving a very good response rate of 80.83%. It is a common agreement amongst AAPD, AAP and ADA that early dental screening is the key to improve infant oral healthcare and prevent ECC.^8,13,14^ The allied healthcare professionals and community organizations must involve and collaborate to achieve this goal. Research study shows that the pediatricians are not advising patients to see the dentist by 1 year of age.^14,15^

Traditionally, AAP had recommended seeing the dentist by the age of 36 months. However, more recently the AAP has changed and expanded its oral health guidelines; and the recent policy aims to establish a dental home for children by 1 year of age through the use of oral health risk assessment at 6 months of age. The policy recommends referring a child for oral health examination within 6 months of eruption of first primary tooth but not later than 12 months of age. The policy also focuses on the specific preventive strategies like diet counseling, optimal use of fluoride and providing anticipatory guidance.^13^ Infant oral healthcare awareness with regard to ECC, oral hygiene practice and specialty treatment to infants rendered by the pediatricians is unclear. Hence, this study was undertaken to know the awareness, attitude and practice of pediatricians toward infant oral healthcare.

Table 1 shows the demographic data of the pediatricians surveyed. Out of the pediatricians surveyed, 67% were males and 33% were females. Since the responses of the questionnaire are qualitative; the correct responses were given a quantitative value and mean score was calculated. The mean score was found to be higher in practitioners with > 10 years of experience as compared to those with < 10 years of practice. The difference in mean score between them was found to be statistically significant (p < 0.001), suggesting that pediatrician with more experience had better knowledge toward infant oral healthcare (Table 1A).

**Table 1:** **Table 1: **Demographic information of the respondents

		*Years of experience*											
		*< 10 years*				*≥ 10 years*				*Total*			
*Gender*		*N*		*%*		*n*		*%*		*n*		*%*	
Male		37		60		28		80		65		67	
Female		25		40		7		20		32		33	
Total		62		100		35		100		97		100	

**Table 1a:** **Table 1A: **Total mean score based on years of experience

*Experience* * (years)*		*n*		*Mean*		*Std.* * dev.*		*Mean* * difference*		*t*		*p-value*	
< 10		62		13.18		3.52		–2.937		–3.924		< 0.001*	
≥ 10		35		16.11		3.57							

All the pediatricians are aware about dentist but only 52% of them refer. Although 93.8% of the pediatricians were aware about the pediatric dentistry as specialty, only 48% referred to pediatric dentist (Table 2). The shortage of pediatric dentists might be the cause for nonreferral. According to the 2011 census, the population of children aged 0 to 6 years in Mysore is 77,988; giving a child to pediatric dentist ratio of 1:3,900, which is quite alarming.^16^ More than 60% of pediatricians in this study were observed to have adequate knowledge about number of primary teeth and age of first tooth eruption (Table 3).

About 77% of pediatricians examine the infants’ oral cavity at birth. Although 82% of pediatricians agree that it is important to do dental examination before 1 year, only 43% of them were aware of the AAPD/AAP recommended first dental visit, and only 11% advised the parents for child’s first dental visit before 1 year of age (Table 4). This could be because many of them are not familiar with recent AAPD/AAP recommendations for pediatric preventive dental care. Some of the pediatricians even question the referral for infants, because previously AAP recommended first dental visit at the age of 36 months. With the increasing incidence of ECC and demand for the total healthcare right from the birth, it is absolutely necessary for the first dental visit below 1 year of age.

The importance of initiating oral hygiene practice before the eruption of first tooth was not seen to be prevalent among the pediatricians. Only 26% of them recommended to clean the oral cavity from the time of birth after every feed and 47% of the pediatricians recommended to clean the teeth using toothbrush and paste only after all the primary teeth erupt (Table 5).

In the US, 98.9% of pediatricians frequently examine a child for signs of dental caries.^4^ In our study, it was found that though 77% of the pediatricians were aware of pediatric dentistry as specialty and the dental problems associated with infants like eruption cyst, Bohn’s nodules and ECC, only 48% of them referred to pediatric dentist (Table 1). Present study revealed that the pediatricians were aware of the factors causing ECC. In all, 94% of the pediatricians examine ECC in infants and 88% of them discuss about ECC with parents. Though 95% of the pediatricians say ‘no to bottle feeding’, only 75% of pediatricians agreed that bottle feeding leads to ECC; and if the child has to be bottle fed at night, 61% of pediatricians advise plain water over milk with sugar. However, 71% of the pediatricians disagree to the fact that even prolonged breastfeeding at night can also lead to ECC. Alhough 95% of pediatricians agree that nutritional counseling is an important aspect of infant oral health care to prevent ECC, only 33% of them implement in their practice (Table 6).

**Table 2:** **Table 2: **Pediatricians’ awareness about dentistry and its specialty branches

Awareness about dentistry		97 (100%)			
Awareness about pediatric dentistry as specialty		91 (93.8%)			
Referral to pediatric 47 (48%) dentist		Referral to dentist		50 (52%)	

**Table 3:** **Table 3: **Pediatricians’ awareness about primary dentition

Number of primary teeth		18 (12%)		20 (69%)		28 (15%)		No response 18 (12%)	
Age of first tooth eruption (months)		4-6 (32%)		6-9 (60%)		9-12 (8%)		No response (0%)	

**Table 4:** **Table 4: **Pediatricians’ awareness about first dental visit

AAP/AAPD recommended age for first dental visit		At birth (15%)		After the eruption of first primary teeth (43%)		After the eruption of all primary teeth (18%)		No response (24%)	
It is important to do dental examination for children < 1 year		Disagree 5 (5%)		Neither agree nor disagree 13 (13%)		Agree 79 (82%)			
Advise parents for the 1st dental visit		Never 14 (14%)		Occasionally/ Sometimes 72 (75%)		Almost every time 11 (11%)			
Examination of oral cavity of infants at birth		Rarely 2 (2%)		Sometimes 10 (10%)		Often 10 (10%)		Always 75 (77%)	

**Table 5:** **Table 5: **Recommendation of oral hygiene practice for infants

Initiation of cleaning of oral cavity		From the time of birth after every feed (26%)		When the first tooth erupts (45%)		After all the primary teeth erupts (22%)		No response (7%)	
Use of tooth paste and brush to clean the teeth		When the first tooth erupts (28%)		After all the primary teeth erupt (47%)		After 5 years of age (10%)		No response (14%)	

**Table 6:** **Table 6: **Awareness, attitude and practice toward ECC in infants

Do you advise bottle feeding for infants		Yes 5 (5%)		No 92 (95%)							
Do you exam ECC in infants		Yes 91 (94%)		No 6 (6%)											
Do you discuss ECC with parents		Yes 85 (88%)		No 12 (12%)											
If bottle fed at night, contents advised in the bottle		Plain water 59 (61%)		Sweetened water 2 (2%)		Fruit juice 2 (2%)		Milk with sugar 16 (16%)		No response 18 (19%)	
Bottle feeding at night leads to dental caries		Strongly disagree 0 (0%)		Disagree 6 (6%)		Neither agree nor disagree 18 (19%)		Agree 38 (39%)		Strongly agree 35 (36%)	
Prolonged breastfeeding leads to dental caries		Strongly disagree 32 (33%)		Disagree 37 (38%)		Neither agree nor disagree 20 (21%)		Agree 5 (5%)		Strongly agree 3 (3%)	
Is nutritional counseling to prevent ECC offered in your practice?		Never 2 (2%)		Rarely 21 (22%)		Sometimes 32 (33%)		Often 25 (26%)		Always 17 (18%)	
Type of nutritional counseling given to prevent ECC		Reduce high sugar snacks 22 (23%)		Reduce high sugar drinks 20 (21%)		Recommending food rich in minerals and vitamins 32 (33%)		All of them 23 (25%)		Others 0 (0%)	
Nutritional counseling is an important aspect of infant oral healthcare to prevent ECC		Strongly disagree 1 (1%)		Disagree 2 (2%)		Neither agree or disagree 2 (2%)		Agree 53 (55%)		Strongly agree 39 (40%)	

Most of the children under 1 year of age are not routinely seen by the dentist, but pediatricians encounter them at least five times before the child is 1 year old.^17^ Hence, pediatricians are in the position to provide information about oral healthcare to parents and make referral to the dentist when required. Studies have shown that if proper awareness is created among pediatricians, they can educate the parents and accurately identify patients in need of referral.^4^ In the present study, 99% of the pediatricians agreed that both medical and dental professionals together are responsible for infant oral healthcare (Table 7). They should work together to appropriately educate and train themselves to be able to provide risk assessment and preventive oral health services.

**Table 7:** **Table 7: **Who is responsible for infant oral healthcare?

Pediatrician		0	
Pediatric dentist		1 (1%)	
Both		96 (99%)	

### Limitations of the Study

Like any other survey, our study had also certain limitations. Since the study was a close ended questionnaire survey, we were unable to obtain their complete awareness on infant oral healthcare. This study was limited to the group of pediatricians to one area and is not representative of the country. Also, the study did not directly address the barrier to access infant oral healthcare.

## CONCLUSION

From this study it can be concluded that majority of the pediatrician are not advising parents to visit dentist by 1 year of age. Many pediatricians feel that knowledge imparted in medical schools is inadequate as far as infant oral health is concerned. There is a need to educate everyone on infant oral healthcare by conducting regular health educative programs. More interactions between the pediatricians and pediatric dentist would be worth to handle the infant population and provide them complete overall health.
